# Generation of Artificial FASTQ Files to Evaluate the Performance of Next-Generation Sequencing Pipelines

**DOI:** 10.1371/journal.pone.0049110

**Published:** 2012-11-12

**Authors:** Matthew Frampton, Richard Houlston

**Affiliations:** Division of Genetics and Epidemiology, Institute of Cancer Research, Surrey, United Kingdom; J. Craig Venter Institute, United States of America

## Abstract

Pipelines for the analysis of Next-Generation Sequencing (NGS) data are generally composed of a set of different publicly available software, configured together in order to map short reads of a genome and call variants. The fidelity of pipelines is variable. We have developed *ArtificialFastqGenerator*, which takes a reference genome sequence as input and outputs artificial paired-end FASTQ files containing Phred quality scores. Since these artificial FASTQs are derived from the reference genome, it provides a gold-standard for read-alignment and variant-calling, thereby enabling the performance of any NGS pipeline to be evaluated. The user can customise DNA template/read length, the modelling of coverage based on GC content, whether to use real Phred base quality scores taken from existing FASTQ files, and whether to simulate sequencing errors. Detailed coverage and error summary statistics are outputted. Here we describe *ArtificialFastqGenerator* and illustrate its implementation in evaluating a typical bespoke NGS analysis pipeline under different experimental conditions. *ArtificialFastqGenerator* was released in January 2012. Source code, example files and binaries are freely available under the terms of the GNU General Public License v3.0. from https://sourceforge.net/projects/artfastqgen/.

## Introduction

Completion of the human genome project (HGP) coupled with developments in sequencing technologies has allowed rapid sequencing of complete human genomes. Most next-generation sequencing (NGS) systems are broadly based on fragmentation of genomic DNA with the oversampling of reads providing the necessary linking information for whole-genome assembly algorithms.

One source of error in variant-calling is inherent sequencing error in NGS technology (i.e. incorrect base calls in the reads). These errors are more likely towards the ends of reads, and in the context of certain sequence motifs [1]. Read alignment and subsequent variant-calling is performed by a NGS analysis pipeline, typically composed of a set of publicly-available software configured together. The performance of pipelines is variable, hence it is highly desirable to evaluate the proficiency of any pipeline under different conditions (e.g. low versus high sequencing error rate, low versus high coverage), and to identify regions of the genome which are likely to be problematic or refractory to sequencing. To facilitate benchmarking of NGS pipelines we have developed *ArtificialFastqGenerator* software.

NGS instruments such as the Illumina Genome Analyzer output reads of nucleotide sequences and corresponding base quality scores, the FASTQ format being the standard text-based representation for these data (see Text S1). *ArtificialFastqGenerator* takes a reference genome as input and outputs artificial FASTQ files. The user can customise DNA template/read length, gap size between paired-end reads, the modelling of coverage based on GC content, whether to use Phred base quality scores taken from existing FASTQ files, and whether to simulate sequencing errors. Since these artificial FASTQs are derived from the reference genome, the reference genome provides a gold-standard for the variant-calling, enabling evaluation of any pipeline.

*ArtificialFastqGenerator* offers advantages over other bioinformatic software tools which generate artificial Illumina reads such as *ART* [2], *WgSim* from the Samtools package [3], *Mason* [4], *SimSeq* [5] and *pIRS* [6]. Firstly, *ArtificialFastqGenerator* provides greater flexibility with respect to modelling coverage based on GC content, because the user can assign relevant parameters, rather than them being learned from resequencing data and hence fixed. Secondly, the software produces detailed coverage and error summary statistics (both regional and overall for coverage). Another distinctive feature is the ability to use real Phred scores taken from existing FASTQ files. By contrast *pIRS* uses an empirical model which predicts the base-call and Phred score based on read cycle, reference base and quality of the previous cycle.

Here, we describe *ArtificialFastqGenerator* and an evaluation of a typical bespoke NGS analysis pipeline. We investigate how variant-calling results are affected by Phred quality scores and simulated sequencing error in the FASTQs, the presence/absence of different stages in the pipeline, and variant-caller choice.

## Artificial Fastq Generator

*ArtificialFastqGenerator* is platform-independent software written in Java SE 6, and is available as open-source from the *SourceForge* website: https://sourceforge.net/projects/artfastqgen/. The program takes a reference genome sequence in FASTA format as input and outputs artificial paired-end FASTQ files [7] which contain Phred base quality scores encoded in the Sanger format (see Text S1 for more description of the FASTQ format).

This section mentions user parameters for controlling DNA template/read length, target coverage, whether to take the base quality scores from existing FASTQ files, and whether to simulate sequencing errors based on these scores. Text S2 describes how to set these parameters and run a test case. In addition to FASTQ files, *ArtificialFastqGenerator* produces a log file of summary statistics for coverage and error generation, and a file documenting start and end indexes in the reference genome of all generated reads.

### Read Generation

We can consider the reference as a sequence of nucleobases stretching from left to right. In order to maintain a small memory footprint, *ArtificialFastqGenerator* generates reads for reference nucleobases which are within a sliding window. The “nucleobaseBufferSize” parameter determines the window's size, and hence also how far right it moves on each occasion.

*ArtificialFastqGenerator* passes through the region inside the window repeatedly, considering pairs of nucleobase sequences for the generation of paired-end reads. Nucleobase *target coverage* constraints dictate that eventually, the program will complete a pass through the region without being able to generate any novel reads, and at this point, the window slides right. A nucleobase's coverage is the number of reads in which it appears, so its target coverage is the upper limit on how many reads it can appear in.

*ArtificialFastqGenerator* can only generate right-end reads for the nucleobases at the right end of the window. Hence when the window slides right, it reaches a point at which these nucleobases are at the left end of the window. This then allows left-end reads including these nucleobases to be generated.

### Read length and Gap Size between Paired-end Reads

In paired-end sequencing both ends of a DNA template are sequenced. If read lengths are constant, which is generally the case, then the distance between the two reads is determined by the template length. *ArtificialFastqGenerator* samples template lengths from a normal distribution. The user can specify the mean and standard deviation of this distribution, and the read length.

### Specifying a Nucleobase’s Target Coverage

To set a nucleobase's target coverage, *ArtificialFastqGenerator* calculates the region's GC content, and then defines and samples from a normal distribution of coverage levels for regions with this GC content. The software calculates the distribution's mean using a Gaussian function of the GC content. The user can customise the function by setting the coverage mean peak (the height of the bell's peak), the GC content at which this peak occurs (the position of the centre of the peak), and how quickly mean coverage decays (the width of the bell). The user can also specify the standard deviation as a multiple of the mean, and the size of the region for which GC content is calculated.

**Figure 1:**
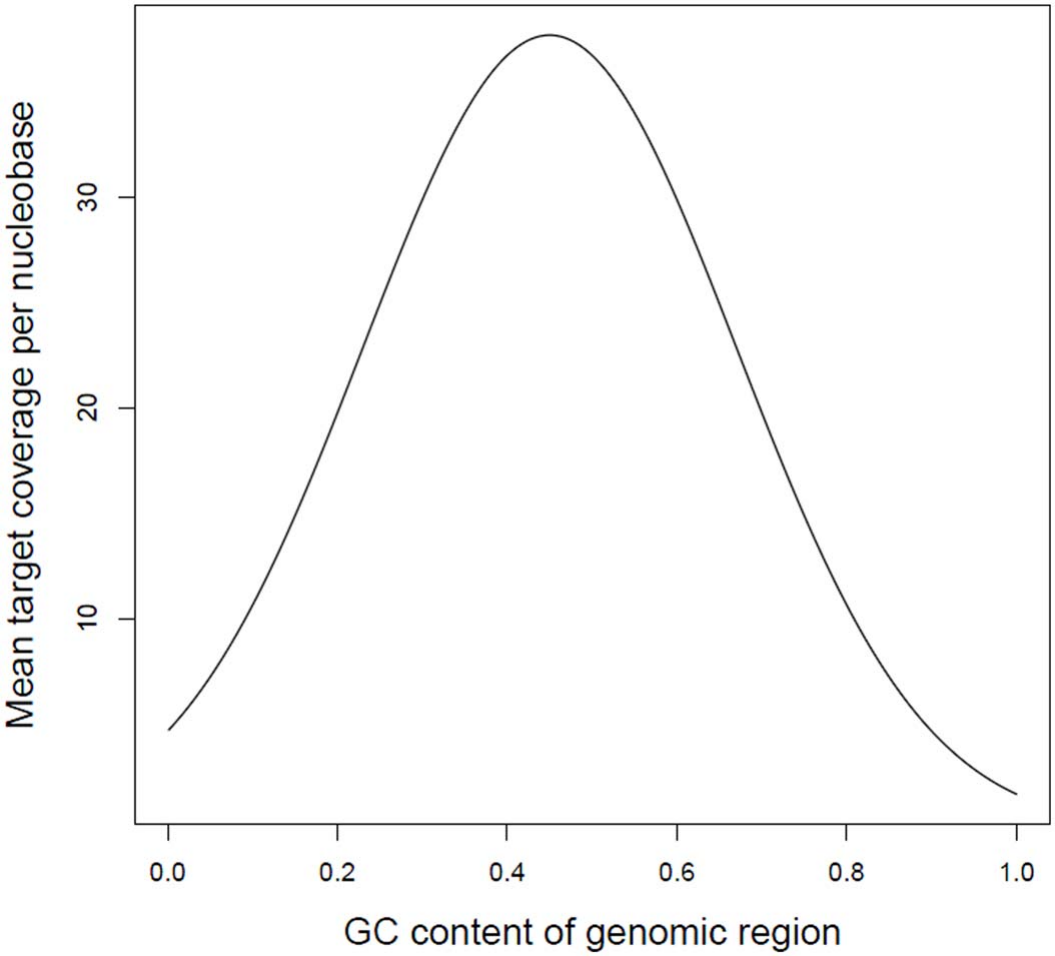
The profile for regional GC content versus mean target coverage, produced by using the default settings for the relevant *ArtificialFastqGenerator* user parameters.

The default values of these parameters are based on the results of a capture probe experiment in which 4 samples were sequenced by an Illumina Genome Analyzer [8]. Figure 1 shows the expected profile for GC content versus mean target coverage when using these default settings. If a different profile better suits the user's needs, then they can change the settings accordingly. There is also a user parameter for switching off the biasing of coverage based on GC content.

### Phred Quality Score and Error Generation

As the default, *ArtificialFastqGenerator* assigns every base in every read a high Phred quality score of 40, Sanger-encoding “I”.Alternatively the program can use quality scores from pre-existing FASTQ files. If a generated read is a different length to the one whose quality scores are being used, then the sequence of quality scores is lengthened/shortened accordingly by either duplicating or removing the first base quality score(s). By only altering the beginning of the sequence, the trend for quality scores to deteriorate at the end of reads is preserved.

The error simulator decodes the base's Sanger format encoded Phred quality score (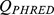), calculating the estimated probability of error (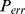) from
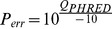
(1)


In the current incarnation of *ArtificialFastqGenerator* the quality score is the sole parameter used for error generation in the program.

Unknown bases (Ns) cannot be subjected to error simulation, and are always assigned a very low Phred quality score of 2. There is a user parameter for filtering out reads which contain Ns.

### Summary Coverage and Error Statistics

*ArtificialFastqGenerator* logs regional and overall summary coverage statistics, and overall error statistics. The overall error statistics include the total number of reads and nucleobase calls, and the number of these nucleobase calls for which an error was simulated.

### Speed

*ArtificialFastqGenerator* has a low memory footprint dependent on the user-specified “nucleobaseBufferSize” parameter. On an Intel Xeon X5650 processor (12 M cache, 2.66 GHz, 6.40 gigatransfers per second Intel QuickPath Interconnect), when using real quality scores and simulating sequencing errors, and with the other parameters set to their defaults, it takes around 6 hrs 20 mins to process 1 giga base.

## Analysis

To illustrate the use of *ArtificialFastqGenerator* FASTQ files to benchmark the performance of a typical NGS pipeline we subjected to scrutiny our current in-house bioinformatic pipeline which is based on the following components:

1. Quality control of raw sequences (FASTQ file reads) using *FastQC* [9], which reports overall GC content, mean base quality score for each position in the reads, distribution of the reads' mean base quality score, and frequency of bases in each read position which are A/C/G/T/N (unknown).
2. Alignment of reads using *Stampy* [10] which outputs files in the Sequence Alignment/Map (SAM) format [3].
3. Local realignment of reads using the *Genome Analysis Toolkit (GATK)* [11], [12]. Local realignment serves to transform regions with misalignments due to InDels, generating a “realigned” BAM file.
4. Marking/removal of Polymerase Chain Reaction (PCR) duplicates using *Picard* [13].
5. Recalibration of base quality scores using GATK.
6. Variant-calling using GATK or alternatively Platypus [14]. Each variant is reported in a *Variant Call Format (VCF)* file along with features which are indicative of its fidelity e.g. mapping quality of the reads, and whether the variation is seen in only one strand. Filtering based on these features can reduce the number of false-positives (FPs).

To evaluate this NGS pipeline we investigated how variant-calling is influenced by: Phred quality scores; Simulated sequencing errors; Local realignment and recalibration; Variant-caller software (GATK vs. Platypus); Coverage and sequence uniqueness.

### Methodology

Using *ArtificialFastqGenerator* and the human reference genome (build 37 in FASTA format), we generated two pairs of whole genome FASTQs with different Phred quality score/simulated sequencing error (PE) characteristics: high Phred quality scores (40) with no simulated sequencing errors (PE = 00); Phred quality scores from pre-existing FASTQ files with simulated sequencing errors (PE = 11). All other parameters in *ArtificialFastqGenerator* were set to their default values (see Text S2). FASTQ files which were used to supply real Phred quality scores were derived from exomes sequenced using an Illumina HiSeq 2000 system.

To speed up the analysis we generated individual chromosome FASTQs in parallel and then merged them. For each FASTQ file, the number of reads was approximately 562,570,000 and GC content 41%–42%, while for each pair, the average coverage per nucleobase was 29.90 to 2 d.p.s (range 0 to 40). Note that this average is for nucleobases marked as A/C/G/T in the reference genome, not N (unknown). Using the Phred quality scores from the Illumina HiSeq FASTQ data resulted in a simulated sequencing error for approximately 4.5% of all nucleobase calls.

To investigate the effect of Phred quality scores and simulated sequencing errors on variant calling, we analyzed each pair of whole genome FASTQs using our NGS pipeline. To investigate the effect of local realignment and recalibration, we conducted variant-calling immediately after the alignment stage, and to investigate the effect of variant-caller choice, we used Platypus rather than GATK. We did not implement Syzygy [15] as this software is primarily designed for targeted resequencing projects. Note that our variant calling results were filtered. For GATK results, we applied VCF filters as recommended on the Broad Institute wiki: for SNPs, *QualByDepth* < 2.0, *RMS MappingQuality* < 40.0, *FisherStrand* > 60.0, *HaplotypeScore* > 13.0, *MappingQualityRankSumTest* < −12.5, *ReadPosRankSumTest* < −8.0; for InDels, *QualByDepth* < 2.0, *ReadPosRankSumTest* < −20.0, *FisherStrand* > 200.0. Platyus automatically applies a filter for each variant call and records whether it passes in the VCF.

To examine the basis of genomic regions consistently associated with FP variant calls we investigated the effect of coverage and the level of uniqueness of their composite sequences. Our basic hypothesis was that such regions have reduced coverage and/or reduced sequence uniqueness. To study the effect of coverage, we made use of coverage statistics for every 10000 nucleobases in the reference genome outputted by *ArtificialFastqGenerator*, and to study sequence uniqueness, we used the *Broad alignability track* [16].

## Results

Note that FP variant calls are made when PE = 00 and PE = 11. We refer to genomic regions containing FP variant calls as *FP variant regions*.

### Rate of False-positive Variant Call Under different Conditions

Figures S1 and S2 show the number of FP variant calls for each chromosome after filtering under different experimental conditions. A number of trends are apparent, including a positive correlation between the number of FP SNPs and InDels, and Platypus making more FP variant calls than GATK. For example, when PE = 11 and after RDMR, Platypus calls 10390 SNPs and 2381 InDels genome-wide, while GATK calls 2604 and 1244.

Imposing real Phred quality scores and simulating sequencing errors is associated with a reduction in FP variant calls. For example, when PE = 00 (and after RDMR), GATK calls 4306 SNPs and 2100 InDels genome-wide, but when PE = 11, it calls 2604 and 1244. While simulating sequencing errors may cause more misaligned reads, fewer are aligned with high confidence, resulting in fewer FP variant calls.

We are most interested in the importance of Realignment, Duplicate Marking and Recalibration (RDMR) when PE = 11, because this is the much more realistic case. As hoped, RDMR reduces the number of FP SNPs (GATK calls fewer in 23 chromosomes, 2604 versus 2691 genome-wide), but it increases FP InDels (GATK calls more in 23 chromosomes, 1244 versus 953 genome-wide).

### Regions Associated with False-positive Variant Calls Under Different Conditions

While different experimental conditions are associated with an increased/decreased number of FP variant calls, the location of *FP variant regions* seems to remain the same. This point is illustrated by Figures S3, S4, S5, S6, S7, S8, S9, and S10–histograms which show the distribution (after filtering) of GATK's chromosome 1 FP variant calls under different experimental conditions. In general, the peaks keep occurring in the same regions. This same observation was made for the other chromosomes.

### Analysis of Regions Containing False-positive Variant Calls

Table S1 shows coverage statistics for a sample of regions (10000 nucleobases long) on chromosome 1 which produced a relatively high number of FP variant calls (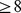). All nucleobases are A/C/G/T (none are N), and PE = 00. A region's average nucleobase coverage ranges from 29.94 to 32.78 (to 2 d.p.s), which is actually higher than genome-wide (29.90 for As/Cs/Gs/Ts), and minimum coverage for a nucleobase is not very low (9–20). This suggests that it is not possible to predict the presence/number of FP SNPs based on coverage alone.

The region in Table S1 with the highest number of FP SNPs is 154420001–154430000. On closer inspection, its FP SNPs seemed to be in 2 clusters–the first, a cluster of 12 in 154421756–154421820 inclusive, and the second, a cluster of 5 in 154428273–154428328. We measured the uniqueness in these FP SNP cluster regions with the *Broad alignability track*, and then also in expanded regions of length 500 and 1000 bases (centered on the original FP cluster region).

Table S2 shows the level of uniqueness statistics in these regions, and also across the whole chromosome. While the first FP SNP cluster region has a low-level of uniqueness, the second does not. Hence, as for coverage, it will not always be possible to predict the presence/number of FP variant calls in a genomic region based only on its level of sequence uniqueness.

## Discussion

Here we have demonstrated that FASTQs generated by *ArtificialFastqGenerator* can be used to evaluate the performance of an NGS analysis pipeline under different conditions and identify which components of a system may be suboptimal. Furthermore, the strategy provides a means of identifying regions of the genome which may be problematic for any pipeline.

Using our own NGS analysis pipeline we investigated how variant-calling is affected by Phred quality scores, sequencing errors, coverage, local realignment and recalibration, and variant-caller algorithm. We found that the number of false-positive variant calls was higher using Platypus rather than GATK for variant-calling, and lower when we used real Phred quality scores and simulated sequencing error. The number of false-positive SNP calls was also lower after local realignment, duplicate marking and recalibration, but unfortunately, the number of false-positive InDels was higher.

Although the number of false-positive variant calls varied under different conditions the regions these were associated with were generally the same. We also established that there is no simple straightforward relationship between the presence or absence of false-positive variant calls, and coverage or sequence uniqueness.

In the current guise *ArtificialFastqGenerator* offers the user control over DNA template/read length, target coverage, whether to use real Phred base quality scores taken from existing FASTQ files, and whether to simulate sequencing errors. Possible future extensions of the software are to offer the user greater choice over quality score generation, and if possible, to improve the accuracy of sequencing error by basing it on more than just the quality scores.

## Supporting Information

Figure S1 **Number of false-positive (FP) variant calls after filtering for chromosomes 1–12; S = SNP; I = InDel; PE = 00 means all Phred scores high (40) & no simulated sequencing errors; PE = 11 means Phred scores from real FASTQ files & simulated sequencing errors; RDMR = local realignment, duplicate marking and recalibration; G = GATK; P = Platypus.**
(TIFF)

Figure S2 **Number of false-positive (FP) variant calls after filtering for chromosomes 13–22, X and Y; S = SNP; I = InDel; PE = 00 means all Phred scores high (40) & no simulated sequencing errors; PE = 11 means Phred scores from real FASTQ files & simulated sequencing errors; RDMR = local realignment, duplicate marking and recalibration; G = GATK; P = Platypus.**
(TIFF)

Figure S3 **GATK chromosome 1 SNP calls for different Phred quality score and sequencing error simulation settings; local realignment, duplicate marking and recalibration (RDMR) are applied, as well as variant-call filtering. PE = 00 means all Phred scores high (40) & no simulated sequencing errors; PE = 11 means Phred scores from real FASTQ files & simulated sequencing errors.**
(TIFF)

Figure S4 **GATK chromosome 1 InDel calls for different Phred quality score and sequencing error simulation settings; local realignment, duplicate marking and recalibration (RDMR) are applied, as well as variant-call filtering; PE = 00 means all Phred scores high (40) & no simulated sequencing errors; PE = 11 means Phred scores from real FASTQ files & simulated sequencing errors.**
(TIFF)

Figure S5 **GATK chromosome 1 SNP calls for different Phred quality scores and sequencing error simulation settings; local realignment, duplicate marking and recalibration (RDMR) are applied but no variant-call filtering. PE = 00 means all Phred scores high (40) & no simulated sequencing errors; PE = 11 means Phred scores from real FASTQ files & simulated sequencing errors.**
(TIFF)

Figure S6 **GATK chromosome 1 InDel calls for different Phred quality score and sequencing error simulation settings; local realignment, duplicate marking and recalibration (RDMR) are applied, but no variant-call filtering. PE = 00 means all Phred scores high (40) & no simulated sequencing errors; PE = 11 means Phred scores from real FASTQ files & simulated sequencing errors.**
(TIFF)

Figure S7 **GATK chromosome 1 SNP and InDel calls when all Phred quality scores high (40) & no simulated sequencing error (PE = 00); local realignment, duplicate marking and recalibration (RDMR) are applied, and also variant-call filtering.**
(TIFF)

Figure S8 **GATK chromosome 1 SNP and InDel calls when using real Phred quality scores and simulated sequencing errors (PE = 11); local realignment, duplicate marking and recalibration (RDMR) are applied, and also variant-call filtering.**
(TIFF)

Figure S9 **GATK chromosome 1 SNP calls after applying local realignment, duplicate marking and recalibration (RDMR) versus not; real Phred quality scores and simulated sequencing errors are used (PE = 11), and variant-call filtering is applied.**
(TIFF)

Figure S10 **GATK versus Platypus chromosome 1 SNP calls when using real Phred quality scores and simulated sequencing errors (PE = 11); local realignment, duplicate marking and recalibration (RDMR) are applied, as well as variant-call filtering.**
(TIFF)

Table S1
**Coverage statistics for a sample of regions from chromosome 1 of length 10000 bases which produced a relatively high number of FP variant calls (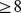); FP = false-positive; PE = 00.**
(PDF)

Table S2 **Broad alignability track results for 2 regions containing a cluster of FP SNPs on chromosome 1, and for chromosome 1 as a whole.**
(PDF)

Text S1 **Short description of the FASTQ format.**
(PDF)

Text S2 **Description of** ***ArtificialFastqGenerator's*** **user parameters, and how to run a test case.**
(PDF)
