# Supplementary figures and images for: Generation of Artificial FASTQ Files to Evaluate the Performance of Next-Generation Sequencing Pipelines
Source: PLoS One. 2012 Nov 12;7(11):e49110. doi: 10.1371/journal.pone.0049110 (PMC3495771; doi:10.1371/journal.pone.0049110)

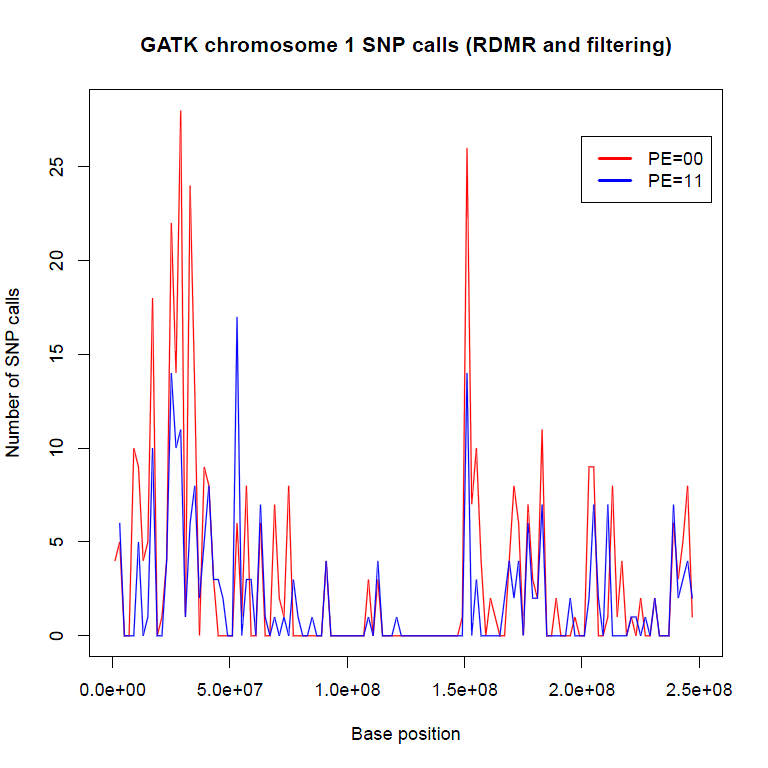

Supplement: Figure S3 — GATK chromosome 1 SNP calls for different Phred quality score and sequencing error simulation settings; local realignment, duplicate marking and recalibration (RDMR) are applied, as well as variant-call filtering. PE = 00 means all Phred scores high (40) & no simulated sequencing errors; PE = 11 means Phred scores from real FASTQ files & simulated sequencing errors. (TIFF) [file pone.0049110.s003.tiff]

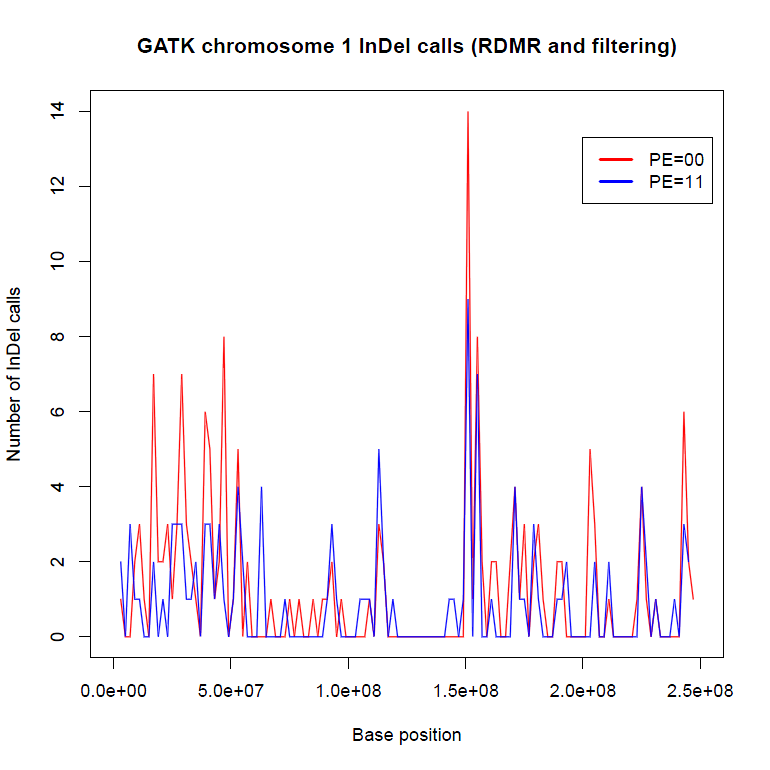

Supplement: Figure S4 — GATK chromosome 1 InDel calls for different Phred quality score and sequencing error simulation settings; local realignment, duplicate marking and recalibration (RDMR) are applied, as well as variant-call filtering; PE = 00 means all Phred scores high (40) & no simulated sequencing errors; PE = 11 means Phred scores from real FASTQ files & simulated sequencing errors. (TIFF) [file pone.0049110.s004.tiff]

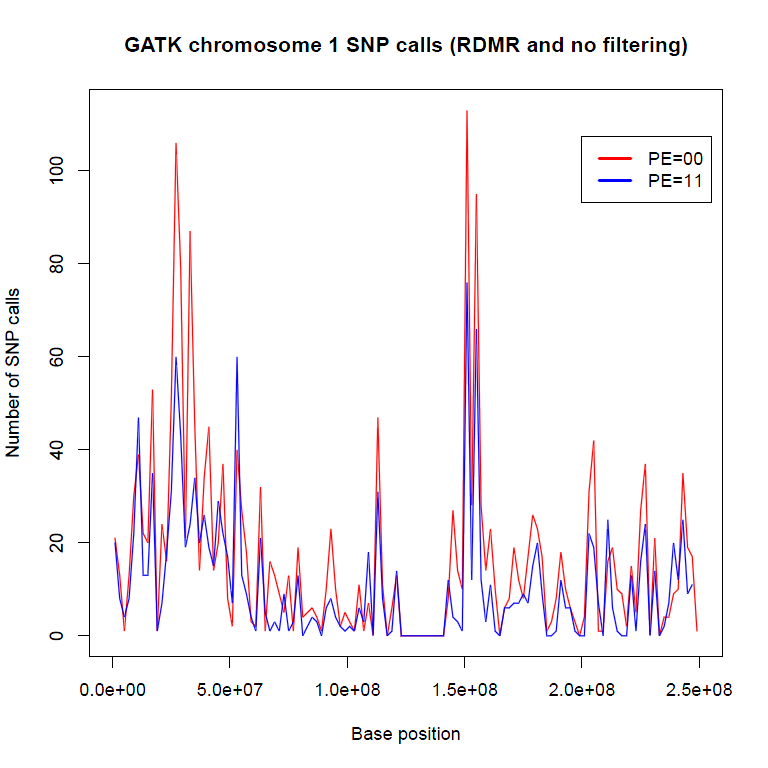

Supplement: Figure S5 — GATK chromosome 1 SNP calls for different Phred quality scores and sequencing error simulation settings; local realignment, duplicate marking and recalibration (RDMR) are applied but no variant-call filtering. PE = 00 means all Phred scores high (40) & no simulated sequencing errors; PE = 11 means Phred scores from real FASTQ files & simulated sequencing errors. (TIFF) [file pone.0049110.s005.tiff]

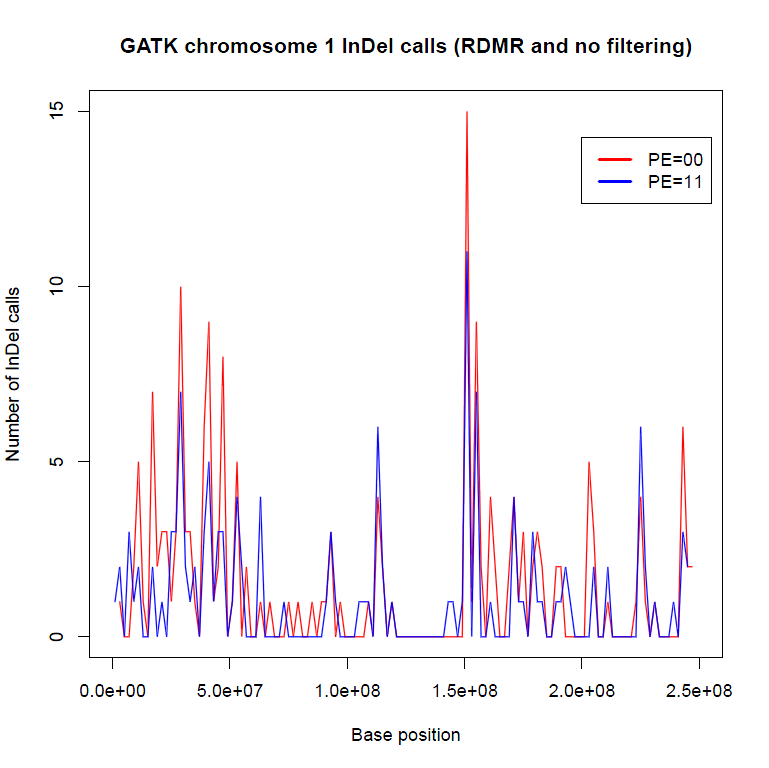

Supplement: Figure S6 — GATK chromosome 1 InDel calls for different Phred quality score and sequencing error simulation settings; local realignment, duplicate marking and recalibration (RDMR) are applied, but no variant-call filtering. PE = 00 means all Phred scores high (40) & no simulated sequencing errors; PE = 11 means Phred scores from real FASTQ files & simulated sequencing errors. (TIFF) [file pone.0049110.s006.tiff]

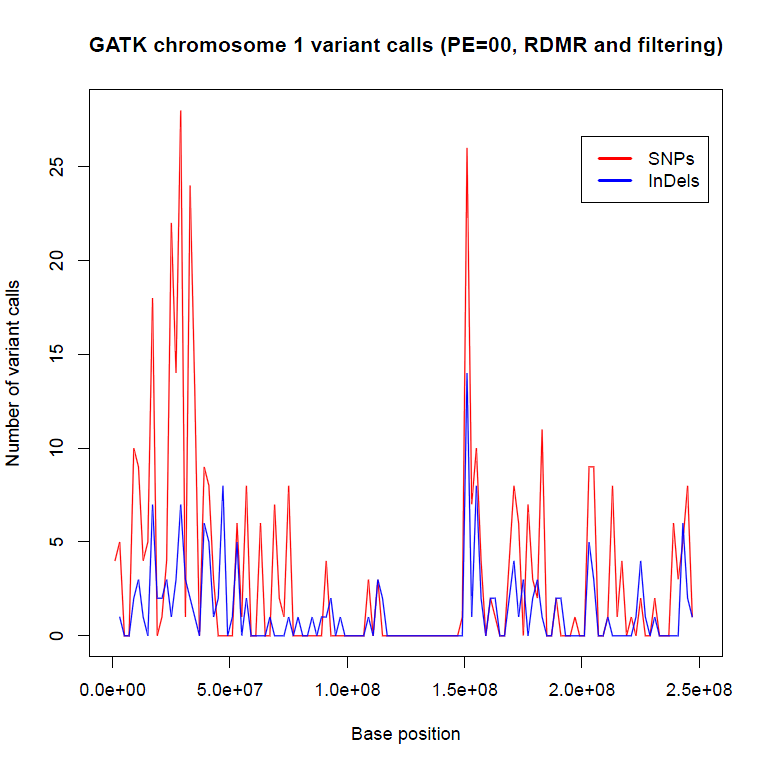

Supplement: Figure S7 — GATK chromosome 1 SNP and InDel calls when all Phred quality scores high (40) & no simulated sequencing error (PE = 00); local realignment, duplicate marking and recalibration (RDMR) are applied, and also variant-call filtering. (TIFF) [file pone.0049110.s007.tiff]

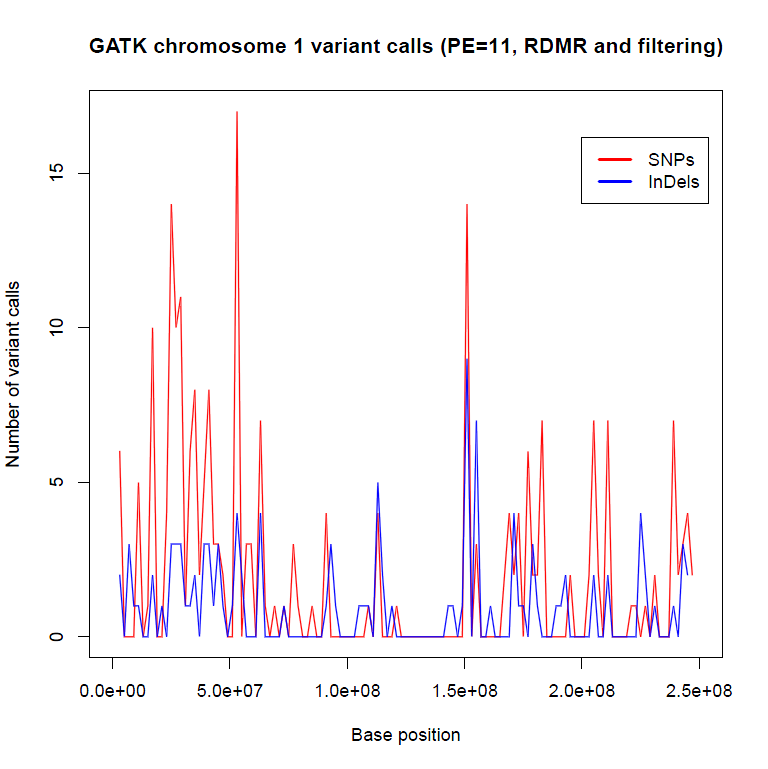

Supplement: Figure S8 — GATK chromosome 1 SNP and InDel calls when using real Phred quality scores and simulated sequencing errors (PE = 11); local realignment, duplicate marking and recalibration (RDMR) are applied, and also variant-call filtering. (TIFF) [file pone.0049110.s008.tiff]

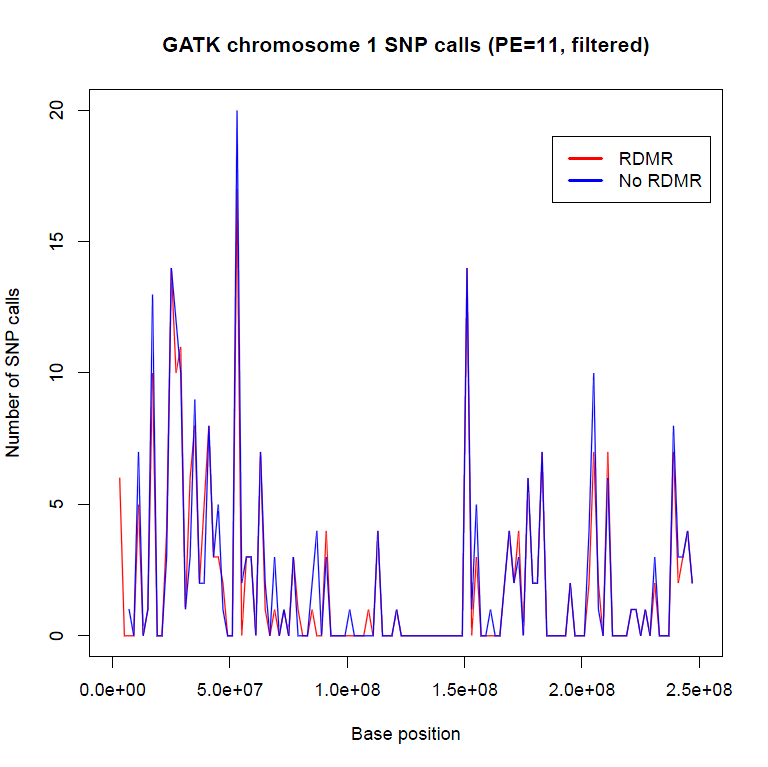

Supplement: Figure S9 — GATK chromosome 1 SNP calls after applying local realignment, duplicate marking and recalibration (RDMR) versus not; real Phred quality scores and simulated sequencing errors are used (PE = 11), and variant-call filtering is applied. (TIFF) [file pone.0049110.s009.tiff]

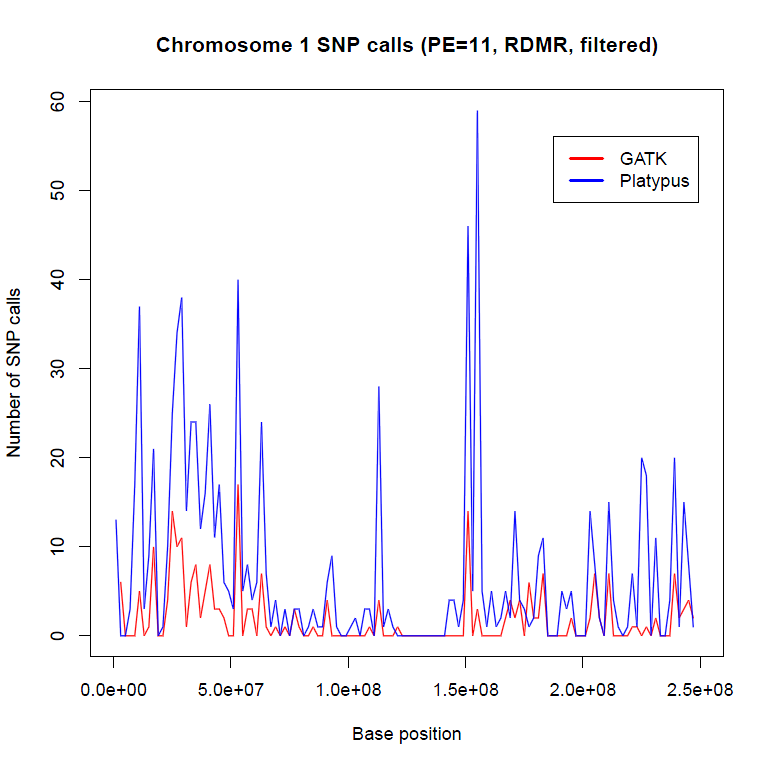

Supplement: Figure S10 — GATK versus Platypus chromosome 1 SNP calls when using real Phred quality scores and simulated sequencing errors (PE = 11); local realignment, duplicate marking and recalibration (RDMR) are applied, as well as variant-call filtering. (TIFF) [file pone.0049110.s010.tiff]
