# Supplementary material for: Generation of Artificial FASTQ Files to Evaluate the Performance of Next-Generation Sequencing Pipelines
Source: PLoS One. 2012 Nov 12;7(11):e49110. doi: 10.1371/journal.pone.0049110 (PMC3495771; doi:10.1371/journal.pone.0049110)
Supplement: Table S1 — Coverage statistics for a sample of regions from chromosome 1 of length 10000 bases which produced a relatively high number of FP variant calls (); FP = false-positive; PE = 00. (PDF) [file pone.0049110.s011.pdf]

Table S1. Coverage statistics for chromosome 1 regions in which there were a relatively high number of false-positive variant calls

Region start and end base positions	Average coverage for a nucleobase	Minimum coverage for a nucleobase	Maximum coverage for a nucleobase	# FP SNPs
150150001-60000	32.287	20	39	8
150200001-10000	29.94	9	38	14
150240001-50000	30.84	13	38	13
150550001-60000	30.82	10	39	10
151230001-40000	32.78	17	38	8
151460001-70000	32.04	12	38	8
153890001-900000	30.83	11	38	8
154180001-90000	31.84	18	38	10
154250001-60000	31.52	13	38	14
154420001-30000	31.67	15	39	17
155540001-50000	31.58	17	38	15

Table 1: Coverage statistics for a sample of regions from chromosome 1 of length 10000 bases which produced a relatively high number of FP variant calls (≥ 8). FP=false-positive; PE=00.
