# Supplementary material for: Generation of Artificial FASTQ Files to Evaluate the Performance of Next-Generation Sequencing Pipelines
Source: PLoS One. 2012 Nov 12;7(11):e49110. doi: 10.1371/journal.pone.0049110 (PMC3495771; doi:10.1371/journal.pone.0049110)
Supplement: Table S2 — Broad alignability track results for 2 regions containing a cluster of FP SNPs on chromosome 1, and for chromosome 1 as a whole. (PDF) [file pone.0049110.s012.pdf]

Table S2. Broad alignability track results

# Bases in view	Region bps	# Data values	Min	Max	Range	Mean	Var	SD
65	154421756-820	2	0	0	0	0	0	0
500	154421538-2037	20	0	0	0	0	0	0
1000	154421288-2287	40	0	1	1	0.25	0.19	0.44
55	154428273-328	3	1	1	0	1	0	0
500	154428051-550	20	0	1	1	0.9	0.09	0.31
1000	154427801-8800	40	0	1	1	0.7	0.22	0.46
247249719	1-247249701	9889989	0	1	1	0.70	0.21	0.46

Table 1: Broad alignability track results for 2 regions containing a cluster of FP SNPs on chromosome 1, and for chromosome 1 as a whole.
