# Supplementary material for: Generation of Artificial FASTQ Files to Evaluate the Performance of Next-Generation Sequencing Pipelines
Source: PLoS One. 2012 Nov 12;7(11):e49110. doi: 10.1371/journal.pone.0049110 (PMC3495771; doi:10.1371/journal.pone.0049110)
Supplement: Text S1 — Short description of the FASTQ format. (PDF) [file pone.0049110.s013.pdf]

# The FASTQ format

There are different variants of the FASTQ format–the original Sanger standard and the Solexa/Illumina variants. Here we focus on the Sanger format because this has found the broadest acceptance and is the only format that *ArtificialFastqGenerator* currently produces. Future work will involve extending the program to produce other formats. The Solexa/Illumina variants differ with respect to the quality scores, details of which can be found in [1].

Below is an example of how a read is represented in a FASTQ file:

```
@HWI-ST745_0097:7:1101:1201:1000#0/1
ACCCTAACCCTAACCCTAACCCTAACCCTAACCCTAACCCTAACCCTAACCCTAACCCAACCCTAACCCTAACCCT
+HWI-ST745_0097:7:1101:1201:1000#0/1
HHHHHHHHHHHHHHHHHHGHHHHHHHHHHHHGFHFHHFHFHEHEEGBGGFHFHHGGBHHEEGGBGFGCDGGGDBGF
```

The first line begins with a '@' character and is followed by a sequence identifier and an optional description. The identifier for a sequence from the Illumina software includes:

1. the unique instrument name (@HWI-ST745_0097 above)
2. flowcell lane (7 above)
3. tile number within the flowcell lane (1101 above)
4. 'x'-coordinate of the cluster within the tile (1201 above)
5. 'y'-coordinate of the cluster within the tile (1000 above)
6. index number for a multiplexed sample, 0 for no indexing (0 above)
7. the member of a pair, /1 for paired-end, /2 for mate-pair (/1 above)

The second line contains the raw sequence letters as ASCII text, and the third begins with a '+' character and is optionally followed by the same sequence identifier as in the first line. The fourth line encodes the base quality values for the sequence in the 2nd line, and so must contain the same number of symbols as letters in the sequence. These quality scores are Phred quality scores encoded as ASCII text. Phred scores are now a de facto standard for representing sequencing base qualities. The Phred software reads DNA sequencing trace files, calls bases and assigns a quality value to each [2, 3]. The Phred quality score of a base call ($Q_{PHRED}$), defined in terms of the estimated probability of error ($P_{err}$) is:

$$Q_{PHRED} = -10 \times log_{10}(P_{err}) \quad (1)$$

Sanger FASTQ files use ASCII 33-126 to encode Phred qualities from 0 to 93. The ASCII encodings are:

```
!"#$%&'()*+,-./0123456789:;<=>?@ABCDEFGHIJKLMNOPQRSTUVWXYZ[\]^_`abcdefghijklmnopqrstuv
wxyz{|}~
```

# References


[1] Cock PJA, Fields CJ, Goto N, Heuer ML, Rice PM (2009) The Sanger FASTQ file format for sequences with quality scores, and the Solexa/Illumina FASTQ variants. Nucleic Acids Research 38: 1767-1771.

[2] Ewing B, Hillier L, C M, Green P (1998) Base-calling of automated sequencer traces using Phred. I. Accuracy Assessment. Genome Research 8: 175-185.

[3] Ewing B, Hillier L, C M, Green P (1998) Base-calling of automated sequencer traces using Phred. I. Error probabilities. Genome Research 8: 186-194.
