# Supplementary material for: Generation of Artificial FASTQ Files to Evaluate the Performance of Next-Generation Sequencing Pipelines
Source: PLoS One. 2012 Nov 12;7(11):e49110. doi: 10.1371/journal.pone.0049110 (PMC3495771; doi:10.1371/journal.pone.0049110)
Supplement: Text S2 — Description of ArtificialFastqGenerator's user parameters, and how to run a test case. (PDF) [file pone.0049110.s014.pdf]

Running *Artificial Fastq Generator*

ArtificialFastqGenerator can be run with any reference sequence in FASTA format. The user parameters are:

- *-h* Print usage help.
- *-O*, <*outputPath*> Path for the artificial fastq and log files, including their base name (must be specified).
- *-R*, <*referenceGenomePath*> Reference genome sequence file, (must be specified).
- *-S*, <*startSequenceIdentifier*> Prefix of the sequence identifier in the reference after which read generation should begin (must be specified).
- *-F1*, <*fastq1ForQualityScores*> First fastq file to use for real quality scores, (must be specified if *useRealQualityScores* = true).
- *-F2*, <*fast2ForQualityScores*> Second fastq file to use for real quality scores, (must be specified if *useRealQualityScores* = true).
- *-CMGCS*, <*coverageMeanGCcontentSpread*> The spread of coverage mean given GC content (default = 0.22).
- *-CMP*, <*coverageMeanPeak*> The peak coverage mean for a region (default = 37.7).
- *-CMPGC*, <*coverageMeanPeakGCcontent*> The GC content for regions with peak coverage mean (default = 0.45).
- *-CSD*, <*coverageSD*> The coverage standard deviation divided by the mean (default = 0.2).
- *-E*, <*endSequenceIdentifier*> Prefix of the sequence identifier in the reference where read generation should stop, (default = end of file).
- *-GCC*, <*GCcontentBasedCoverage*> Whether nucleobase coverage is biased by GC content (default = true).
- *-GCR*, <*GCcontentRegionSize*> Region size in nucleobases for which to calculate GC content, (default = 150).
- *-L*, <*logRegionStats*> The region size as a multiple of -NBS for which summary coverage statistics are recorded (default = 2).
- *-N*, <*nucleobaseBufferSize*> The number of reference sequence nucleobases to buffer in memory, (default = 5000).
- *-OF*, <*outputFormat*> 'default': standard fastq output; 'debug_nucleobases(_nuc||read_ids)': debugging.
- *-RCNF*, <*readsContainingNfilter*> Filter out no "N-containing" reads (0), "all-N" reads (1), "at-least-1-N" reads (2), (default = 0).
- *-RL*, <*readLength*> The length of each read, (default = 76).
- *-SE*, <*simulateErrorInRead*> Whether to simulate error in the read based on the quality scores, (default = false).

- *-TLM*, <*templateLengthMean*> The mean DNA template length, (default = 210).
- *-TLSD*, <*templateLengthSD*> The standard deviation of the DNA template length, (default = 60).
- *-URQS*, <*useRealQualityScores*> Whether to use real quality scores from existing fastq files or set all to the maximum, (default = false).
- *-X*, <*xStart*> The first read's X coordinate, (default = 1000).
- *-Y*, <*yStart*> The first read's Y coordinate, (default = 1000).

Test case

ArtificialFastqGenerator comes with additional files which can be used to test it: *miniReference.fasta*, *test1.fastq* and *test2.fastq*. The *miniReference.fasta* file contains about 100000 nucleobases from each of chromosomes 1 and 2 in the human reference genome, and 120 from chromosome 3, while *test1.fastq* and *test2.fastq* contain 10000 paired-end reads. The command below will generate the paired-end artificial FASTQs for chromosome 1, accepting Phred quality scores from *test1.fastq* and *test2.fastq*, and using them to simulate sequencing errors. The output path should include the file base name (e.g. $OUTPUT_DIR/Chr1), and the -S and -E parameters are prefixes of the desired sequence identifiers, sufficiently long to ensure a match.

java -jar ArtificialFastqGenerator.jar -R miniReference.fasta -O Chr1 -S ">1" -E ">" -URQS true -SE true -F1 test1.fastq -F2 test2.fastq

Apart from the artificial FASTQs, *ArtificialFastqGenerator* also outputs a file which contains the start and end indexes in the reference sequence of all the generated reads, and a log file which contains the parameter settings and summary coverage and error statistics. The user can check the log file and use *FastQC* to confirm that the generated FASTQs have the expected characteristics given the parameter settings.
